# The ‘seet’ danger call: an active nest warning in superb fairy-wrens

**DOI:** 10.1098/rsos.251100

**Published:** 2025-11-26

**Authors:** Lauren K. Common, Alper Yelimlieş, Diane Colombelli-Négrel, Victoria I. Austin, Sonia Kleindorfer

**Affiliations:** ^1^ Konrad Lorenz Research Center, core facility of the University of Vienna, Grünau im Almtal, Upper Austria 4645, Austria; ^2^ Department of Behavioral and Cognitive Biology, University of Vienna, Vienna, Austria; ^3^ College of Science and Engineering, Flinders University, Adelaide, South Australia, Australia; ^4^ Gulbali Institute, Charles Sturt University, Albury, New South Wales, Australia

**Keywords:** vocal communication, antipredator behaviour, reproductive behaviour, passerine, alarm call

## Abstract

Alarm calls are a common, widely studied antipredator behaviour, with species producing different call types that can have distinct functions. Here, we describe a new type of alarm call in the superb fairy-wren (*Malurus cyaneus*), the ‘seet’ danger call. We (i) describe the acoustic characteristics and context of the danger call (in comparison with two other alarm calls); (ii) test its occurrence in relation to predator proximity to nest and nesting stage; and (iii) test the response of birds to experimental broadcast of aerial versus danger calls, given their similar acoustic structure. The danger call is a high-frequency, down-sweep call. Danger calls were produced in all nesting stages, with more calls given when the human threat was closer to the nest. Most danger calls were produced during the feeding phase, and the fewest were produced after fledging. Groups produced more danger calls when responding to experimental broadcasts of danger versus aerial calls. We propose that the ‘seet’ danger call functions as a warning to conspecifics of a threat approaching immobile or moderately mobile and, hence, vulnerable individuals. The occurrence of a nest-based alarm call that covaries with nest content raises the question of which cues adults use to assess offspring vulnerability.

## 1. Introduction

Bird calls, although shorter in duration than songs, can convey information across a variety of contexts and span a broad range of frequency bandwidths and acoustic characteristics [1,2]. Alarm calls, vocalizations produced in the face of danger, are the most widely studied call type and are a common form of antipredator behaviour [3]. Some alarm calls are produced selectively and provide the receivers (conspecifics or heterospecifics) with information about predators, i.e. functional reference [4]. For example, vervet monkeys produce calls that refer to a particular predator or hunting style, such as the ‘eagle’, ‘snake’ and ‘leopard’ calls, which elicit predator-specific antipredator behaviours in the receiver(s) [5,6]. There is evidence for functional reference within alarm calls in many taxa, including in passerines [7,8]. Alarm calls may be highly specific, referring only to a single predator species, or more broad, referring to terrestrial versus avian predator categories [7]. Alarm calls can encode information beyond predator category. The type of alarm call or the number of elements produced may convey details about predator distance [9,10], size [11] and behaviour [12]. For example, New Holland honeyeaters (*Phylidonyris novaehollandiae*) alter the acoustic structure of the first element and change the number of elements in their alarm calls to convey risk level and urgency [13]. In general, to understand the context and function of alarm calls, it is useful to have information about the acoustic structure of the call, the context in which it is given and the behaviour it elicits in the receivers.

Alarm calls often vary in structure and elicit different responses from conspecifics across the breeding season, probably due to shifts in brood value and ontogenetic constraints [14]. Certain call types are produced exclusively during the breeding period and may differ from other calls in both function and intended receivers. In some cases, specific alarm calls function in the context of parent–offspring communication, with parental calls eliciting antipredator responses in nestlings and fledglings [15–18]. For example, in moustached warblers (*Acrocephalus melanopogon*) and Japanese tits (*Parus minor*)*,* nestlings reacted to parental alarm calls with antipredator behaviour, either remaining in the nest in response to aerial predators or jumping from the nest in response to terrestrial predators [15,18]. Southern house wren nestlings (*Troglodytes musculus*) reduced their begging frequency and activity when parents alarm called [19]. Certain alarm calls can also elicit nest defence behaviour in parents. Some species that are hosts of brood parasites, such as the yellow warbler (*Setophaga petechia*), produce specific calls when a cowbird is detected and perform nest defence behaviour in response [20,21], but only in populations with experience with cowbirds [22]. Investigating the variety and function of calls given during reproduction can improve our understanding of nest predator-specific antipredator behaviour.

Superb fairy-wrens (*Malurus cyaneus*) are highly vocal, small, group-living passerines native to scrubland in southeastern Australia [23,24]. Both sexes produce complex chatter songs learnt from both parents [25,26]. Females produce incubation calls from within the nest during the late incubation, whereby embryos learn and produce their (foster) mother’s signature element B (from the incubation call) as their begging call once hatched [27–29]. Males sing a trill song in response to loud predator calls and during the dawn chorus to obtain extra-pair paternity [30,31]. Fairy-wrens also produce multiple documented alarm calls, including terrestrial alarm calls (‘chit’ call, also called the mobbing alarm call, [24,32]), aerial alarm calls (‘flee’ call, [24,33]), brood parasite mobbing calls (‘whining’ call, [34]) and distress calls (produced when an individual is captured, [35]). The urgency of aerial alarm calls, given in bouts, can be increased by increasing the number of calls per bout [33,36]. Under threat of imminent danger, adult superb fairy-wrens produce an ‘alarm song’, distinct from both alarm calls and trill songs [37]. The most urgent alarms are the distress call and the ‘alarm song’. Alarm songs elicit increased and distinct anti-predator behaviour in conspecifics, especially in the partner of the caller [37]. Despite significant study into the vocal communication of fairy-wrens, many calls remain undescribed and with functions unknown [38]. In splendid fairy-wrens (*Malurus splendens melanotus*) and variegated fairy-wrens (*Malurus lamberti*), ‘seet’ calls were given in response to predator vocalizations [38,39]; however, the ‘seet’ call is yet to be documented or described in the superb fairy-wren.

**Figure 1.**
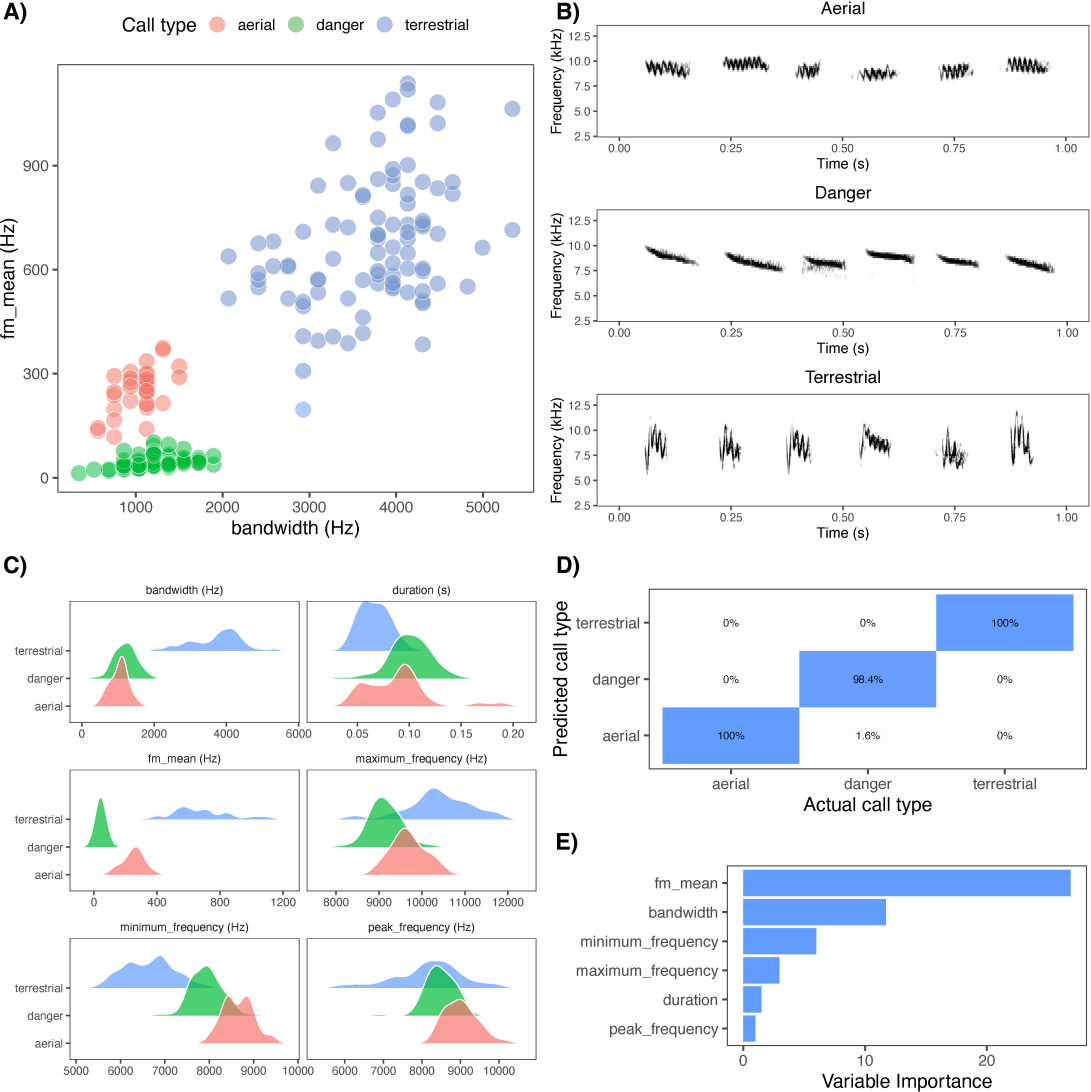
(A) Clustering of call types (aerial, danger and terrestrial alarm calls) by bandwidth (Hz) and frequency modulation (fm_mean; Hz). (B) Spectrograms of superb fairy-wren aerial (first row), danger (second row) and terrestrial alarm calls (third row), created using the package ‘seewave’ in R [40] using a Hanning window with 256 samples per window and 90% overlap. The figure does not reflect natural call rates or bouts but has been modified to facilitate visualization of several different calls within the same call type. (C) Acoustic variables per call type. (D) confusion matrix of discrimination accuracy of call types by random forest classifier. (E) importance of each acoustic variable (minimum frequency, mean frequency modulation, bandwidth, maximum frequency, peak frequency and duration) within the random forest classifier.

During routine monitoring in 2022, we noted that two calls were produced by superb fairy-wrens while a perceived threat, humans, was in close proximity to active nests and young fledglings: the terrestrial alarm call and a single note call, similar in structure to the ‘seet’ call described in other fairy-wren species [38,39]. In this study, we aim to (i) describe the context and acoustic properties of the second call (hereafter referred to as the ‘danger’ call; [37]). (ii) Investigate the relationship between the three main alarm calls (aerial, terrestrial and danger calls) in relation to (a) nest activity or inactivity, (b) predator proximity to the nest, and (c) nesting stage. If danger calls are related to the nest, we predicted more danger calls and a higher proportion of danger calls to aerial and terrestrial alarm calls, when (a) the nest is active, (b) the threat is closer to the nest, and (c) during later nesting stages. If aerial and terrestrial alarm calls are not related to the nest, we predict no change in the number of aerial or terrestrial calls in relation to threat distance to the nest and nesting stage. (iii) Given similarities in the acoustic space occupied by aerial and danger calls (figure 1), and that ‘seet’ calls are often used as aerial alarm calls in other species [41,42], we conducted a playback experiment to determine if individuals differ in their behavioural or vocal response to the ‘seet’ danger versus aerial alarm call types.

## 2. Material and methods

### 2.1. Study site and species

Data were collected in 2023 and 2024 at Cleland Wildlife Park (34°58′ S, 138°41′ E) in South Australia. Superb fairy-wren territories at this site have been continuously monitored since 2008, and most (greater than 90%) individuals are colour-banded (e.g. [25,32]). Superb fairy-wrens are small (approx. 9 g) insectivorous songbirds that occur throughout southeastern Australia [24,43]. Territories often consist of a dominant breeding pair, with related and unrelated helpers (often sons from previous broods; [44–46]). Dominant individuals are distinguished from helpers based on their behaviour, age and breeding activity. During the breeding season (September to January), dominant females are the sole nest builders and incubators, laying a clutch of 2‒4 eggs and incubating for 14 days [24]. The dominant male and helpers assist with feeding the nestlings once they hatch [45]. Nestlings fledge at 10‒14 days but are poor fliers in the first two weeks and remain nutritionally dependent until approximately 40 days old [24].

### 2.2. Nest monitoring

We monitored territories of superb fairy-wrens for nesting activity from September to January in 2023 and 2024 following our long-term monitoring protocol [28,47]. Territories were monitored for group size (2‒4, mean = 2.6 ± 0.2) and the identity of all group members. We monitored dominant females every 3 days for nesting activity. Once found, usually during the building (where the female builds the structure of the nest with sticks, grass, leaves and moss) or lining phase (where the female lines the inside of the nest with fine grass, feathers and fur), we monitored the nest for activity and checked nests every 3 days for egg laying and during incubation and every 2 days during feeding until fledging.

### 2.3. Call recording

We aimed to record all three alarm call types (aerial, danger, terrestrial) at different distances and during each nesting stage (nest lined, incubation, feeding, fledglings and recently inactive). We started at 20 m from the nest, which is generally the edge of the territory as territories are small at our study site (S Kleindorfer *et al.,* personal observation). First, we visually confirmed that the territory was empty of predators and that the group was foraging without alarm or distress. Once certain that at least one member of the group was seen within 10 m, we recorded vocalizations for 1 min, standing still but facing towards the nest. After 1 min of recording, we walked to 15 m and recorded for 1 min. This was repeated for 10, 5, 2 and 0 m (directly next to the nest). If the group moved away, recording was paused and resumed once they returned to ensure they were aware of the distance of the threat to the nest. As the same nest was approached several times over the nesting cycle, we standardized the direction of approach by always approaching from the same direction, which was selected to have a clear and direct 20 m line to the nest. Calls were recorded using a Sennheiser ME67 (Sennheiser electronic GmbH & Co. KG, Germany) directional microphone connected to a Zoom H5 (Zoom Corporation, Japan) recorder. Recordings were imported into Audacity v. 3.4.2 (Muse Group) and number of danger calls and number of other alarm calls (terrestrial, aerial) were counted per minute with a window size of 512 and the window type set to Hanning. We counted each individual call. Generally, individuals could not be seen while giving danger calls (LK Common, personal observation), and so we were unable to quantify the number of calls given per individual, only the number of calls given per territory. All recordings were scored by a single researcher (L.K.C.). We collected 37 recordings from 23 nests in 17 territories.

### 2.4. Acoustic properties

To describe the acoustic properties of the call types (aerial alarm call, danger call, terrestrial alarm call), we selected signals with high quality (not overlapping with any other signal and clearly separable from background noise) by inspecting spectrograms created using Hanning windows with a length of 256 and 50% overlap on Raven Pro 1.6.5 (K. Lisa Yang Center for Conservation Bioacoustics, 2022). We then calculated the fundamental frequency contour for each selection using Raven Pro’s dominant frequency contour function; for all the call types, the dominant and the fundamental frequencies were the same. From the contour, we extracted the call’s minimum and maximum fundamental frequencies (Hz) alongside the mean frequency modulation rate (using the simple spectrographic method described by [48]). We calculated the bandwidth for each call by subtracting the minimum from the maximum fundamental frequency. We also extracted frequency with the highest amplitude from a mean power spectrum of the whole call (peak frequency, Hz) and duration (s) for each call. Overall, we analysed 33 aerial alarm calls, 91 terrestrial alarm calls and 90 danger calls from recordings between 2012 and 2024.

### 2.5. Playback experiment

Playback tracks consisted of 3 min silence (baseline) and 1 min stimulus (playback). We constructed playback tracks from danger calls collected from the group during September–December in 2024 and aerial alarm calls collected from different territories in previous years (2012–2016) as we recorded so few in 2023 and 2024. The aerial alarm calls used were produced in response to a natural aerial predator flying overhead during routine song recording as noted by the observer. We began recording the danger call in 2023 after we became aware of it in 2022, which prompted us to investigate it systematically during 2023 and 2024. Our recordings and analyses of this call type date from 2023, but we recorded new calls in 2024 for the playback stimuli. Aerial call recordings were available from previous years, and previous study has shown that birds respond to aerial alarm calls regardless of kinship or species [33,42]. We edited all stimuli files using Audacity. The peak call amplitude was normalized to −5 dB for each call. The volume of the playback was approximately 70 dB from 1 m away measured using a sound metre (Voltcraft SL-100), to approximate the natural calling amplitude of a fairy-wren [32]. The sound meter was set to A weighting and fast temporal integration. To reduce ambient noise, we applied a high-pass filter at 7 kHz for both call types. The playback phase alternated between 5 s of unique calls derived from the same alarm call type, with one call per second, and 5 s of silence, repeated six times, which reflects a natural calling rate of danger calls (range 9.70 ± 1.42 to 31.60 ± 4.33 calls per minute, see §3). The same five calls were repeated in a random sequence for each of the bouts (e.g. bout one comprised five different calls in the sequence ABCDE, bout two BDECA, bout three DAEBC, etc.). Aerial alarm calls are more often given in bouts [33], with the number of elements per bout encoding urgency [36], and receivers generally respond by fleeing [33,36]. The order of the five calls was randomized using a random number generator for each 5 s bout. As we often could not see individuals while they produced danger calls while recording, we are unable to control for number of individuals within the playback tracks. We constructed 19 danger call tracks (one per experiment per territory) and 11 aerial alarm call tracks. We did not repeat playback stimuli within a territory.

We conducted the experiment between October 2024 and January 2025 between days 7 and 9 of incubation (mean 8 ± 0.2 days) between 08.00 and 16.00 local time. Each group was tested twice: once with danger calls and once with aerial calls. The stimuli were broadcast using a Sony SRS-XB13 speaker placed 3 m from the nest. Once the speaker was placed, we briefly checked the nest to confirm activity and to flush the female if she was incubating. We visually scanned the territory before commencing playback to ensure no obvious predators were present as they may have a confounding effect on the results. We randomized which track (aerial or danger) would be presented first, to control for potential effects of order. Once the first playback track was complete, we collected the speaker and did not enter the territory for at least 1 hour before conducting the second playback. We selected this timeframe between trials to avoid potential differences in activity at different times of the day, and to avoid predation of the nest between trials, as predation rate is high at our study site. Vocal responses were recorded, and behavioural responses of all individuals that approached were narrated into recordings made by a Sennheiser MKE 600 directional microphone (Sennheiser electronic SE & Co. KG, Germany) with a Zoom F3 field recorder (Zoom Corporation, Japan) by experimenters standing more than 10 m from the nest and the speaker. Because individuals were individually marked and multiple observers were present, the behaviour of all responding individuals in a territory was scored. If all members of the group were over 15 m from the speaker at the end of the baseline period, we paused the playback and only restarted once at least one member was within 15 m of the nest or the speaker, to ensure the playback could be heard by the subjects. We narrated the distance of each individual to both the speaker and the nest throughout the baseline (3 min silence) stage and playback stage. The speaker placement in relation to the nest was used as a distance marker. At least two experimenters were present for all experiments, and both experimenters narrated both playbacks simultaneously, but separately. We scored and analysed 1 min, the last minute, of baseline behaviour and 1 min (full duration) of active playback duration. From the recordings, we extracted the time (in seconds) spent within 10 m of the nest (per individual), minimum distance to the nest (in metres, per individual), number of danger calls (group level) and number of terrestrial alarm calls (group level). No aerial alarm calls were produced at any point during the duration of the experiment. We conducted a total of 38 playback experiments (19 aerial and 19 danger) at 12 territories (1–3 experiments per territory, mean 1.6 ± 0.2). We only conducted one experiment per nesting attempt, so all repeat trials were at new nests. We collected both individual-level behavioural responses (*n* = 31 individuals) and group-level vocal responses, as vocal responses could not be consistently attributed to individuals and therefore number of calls were counted for all members of the group.

### 2.6. Ethical statement

This research was approved by the Flinders University Animal Welfare Committee (E480, AEC BIOL5563). Fieldwork was conducted under a permit from the South Australian Department for Environment and Water (Z24699) and the Australian Bird and Bat Banding Scheme (banding authority numbers 2601 and 2719).

### 2.7. Statistical analysis

All statistical analyses were conducted in R v. 4.5.0 [49]. Generalized linear mixed models (GLMMs) were calculated using the ‘glmer’ function in the package lme4 v. 1.1-33 [50]. Continuous variables were scaled to facilitate the interpretation of effect sizes (standardized to mean = 0 and s.d. = 1). Model assumptions were assessed through the package DHARMa v. 0.4.4 [51]. We extracted *χ*^2^ and *p*-values from the ANOVA Table of Deviance using Type III *χ*^2^ tests using the ‘Anova’ function in the package car v. 3.0-12 [52]. We report model effect sizes as estimate ± s.e. using the ‘summary’ function. Predicted values of models were extracted using the ‘ggpredict’ function in the ggeffects v. 1.2.3 [53] and visualized using ggplot2 v. 3.3.5 [54] and ggpubr v. 0.4.0 [55].

#### 2.7.1. Acoustic analysis

We trained a supervised, block cross-validation random forest classifier [56] on six acoustic measures: minimum frequency, maximum frequency, bandwidth, duration, mean frequency modulation rate and peak frequency using the randomForest package v. 4.7-1.2 [57] to assess the discriminability of the call types. Random forest is a machine learning approach that constructs and evaluates decision trees to classify calls of unknown type. For training, we used a random subset of the dataset containing 60% of the territories, then we tested the classifier on the other 40%. We randomly selected territories rather than calls to account for repeated recordings per territory and hence avoid autocorrelation by ensuring that the same territory was not in both the training and the test subsets (block cross-validation [56]). However, territories were not known for aerial alarm calls, and so each recording was assumed to be a separate territory. We report accuracies per call type on the test dataset and variable importance for the classifier. We assessed each variable’s importance for the classification using mean decrease in the Gini Index.

#### 2.7.2. Calls in relation to nest stage and proximity

To determine if the number of danger calls differs between active and inactive nests, we used a Poisson GLMM. We only included the number of danger calls given when the threat, i.e. the human, was directly next to the nest (0 m distance) to isolate the effect of nest activity without distance to nest. Group size was included as a fixed effect to control for its effects. As there were repeated measures per territory, we included territory ID as a random effect.

At active nests, to investigate the effects of threat distance, nesting stage and group size on the number of calls (danger, aerial, terrestrial), we used GLMMs on recordings collected from active nests across four nesting stages (lined, incubating, feeding, fledglings). The dependent variables were (i) the number of danger calls and (ii) the number of terrestrial alarm calls. There were no aerial alarm calls produced during recording so they were not analysed. Both models used a negative binomial distribution to account for overdispersion. Territory ID and recording ID were included as random effects, as some territories were recorded multiple times, and each recording contained multiple threat distances. We used the package ‘emmeans’ v. 1.7.1-1 [58] to calculate pairwise post hoc comparisons with Tukey *p*-value adjustment of significant nesting stage effects. We originally included the interaction between distance and stage (threat distance × stage); however, it was not significant in either model, and its removal improved model fit (as determined by comparing corrected Akaike information criteria calculated using the package AICcmodavg v. 2.3-4 [59]).

To determine if the proportion of danger calls to other alarm calls differs between threat distance, nesting stage and group size, we used a binomial GLMM. The proportion of calls was modelled using the column bind (‘cbind’) function designed to fit proportion data in logistic regression models with the number of danger calls as the binomial denominator, a binomial distribution and a logit link function. We fitted the threat distance, nesting stage, the interaction between threat distance and nesting stage, and group size as fixed effects. We included territory ID and recording ID as random effects.

#### 2.7.3. Playback experiment

We focused on the two call types characterized by longer downward sweeps and higher frequency acoustic features, the aerial alarm call and danger call, to test whether birds acoustically distinguish between them. We conducted four GLMMs to determine if call types (aerial alarm versus danger) affected the individual behavioural response and the group-level vocal response.

The first and second models tested the effect of call type on an individual’s time spent within 10 m of the nest and minimum distance to the nest. We scored an individual’s minimum distance to the nest to the nearest 1 m when within 10 m, and as 11 m when outside of 10 m, resulting in a negatively skewed distribution. Therefore, we converted the minimum distance to a binomial variable, where 0 = minimum distance greater than 10 m and 1 = minimum distance 10 m or less. We used a generalized Poisson model (family = genpois) implemented in glmmTMB v. 1.1.11 [60] to account for underdispersion for the first model, and a binomial distribution for the second model. Call type (aerial/danger), stage (baseline/playback), their interaction (call type × stage), order (whether the call type was presented first or second, categorical variable) and group size were included as fixed effects. Individual ID, territory ID and trial ID were included as random effects to account for repeated measures of individuals and territories within and across playback experiments.

Next, we ran three GLMMs to determine the effect of call type on the number of (i) aerial alarm calls, (ii) danger calls, and (iii) terrestrial alarm calls produced by the group. However, no aerial calls were produced, and hence this call type was not considered further (see §3). For the number of danger calls, a Poisson error distribution was assumed. For the number of terrestrial alarm calls, a negative binomial distribution was assumed to account for overdispersion. Call type (aerial/danger), stage (baseline/playback), their interaction (call type × stage), order (1/2) and group size were included as fixed effects in both models. Territory ID and trial ID were included as random effects to account for repeated testing of territories.

## 3. Results

### 3.1. Call description

Danger calls were high-pitched down-sweep vocalizations with a relatively narrow bandwidth (approx. 8–9 kHz) (figure 1). Aerial alarm calls occupied a similar frequency range (approx. 8–9 kHz) (figure 1). Both aerial and terrestrial alarm calls were more frequency modulated than danger calls; compared with aerial calls, terrestrial alarm calls exhibited rapid frequency modulation over a broader range (approx. 1–6.5 kHz; see figure 1). Terrestrial alarm and danger calls shared similar peak frequencies and durations (approx. 8 kHz), while aerial alarm calls had a slightly higher peak frequency (approx. 9 kHz). Both aerial and terrestrial alarm calls were typically given in bouts of two or more, whereas danger calls were produced singly, with each danger call separated by at least approximately 0.5 s (compared with the separation of approx. 0.05 s between terrestrial calls within bouts).

Using six acoustic parameters, the random forest classifier classified call types with 99% accuracy (figure 1D). Frequency modulation and bandwidth were the most informative for call classification, followed by minimum frequency (figure 1E). Peak frequency was the least informative variable.

### 3.2. Danger call context

**Figure 2.**
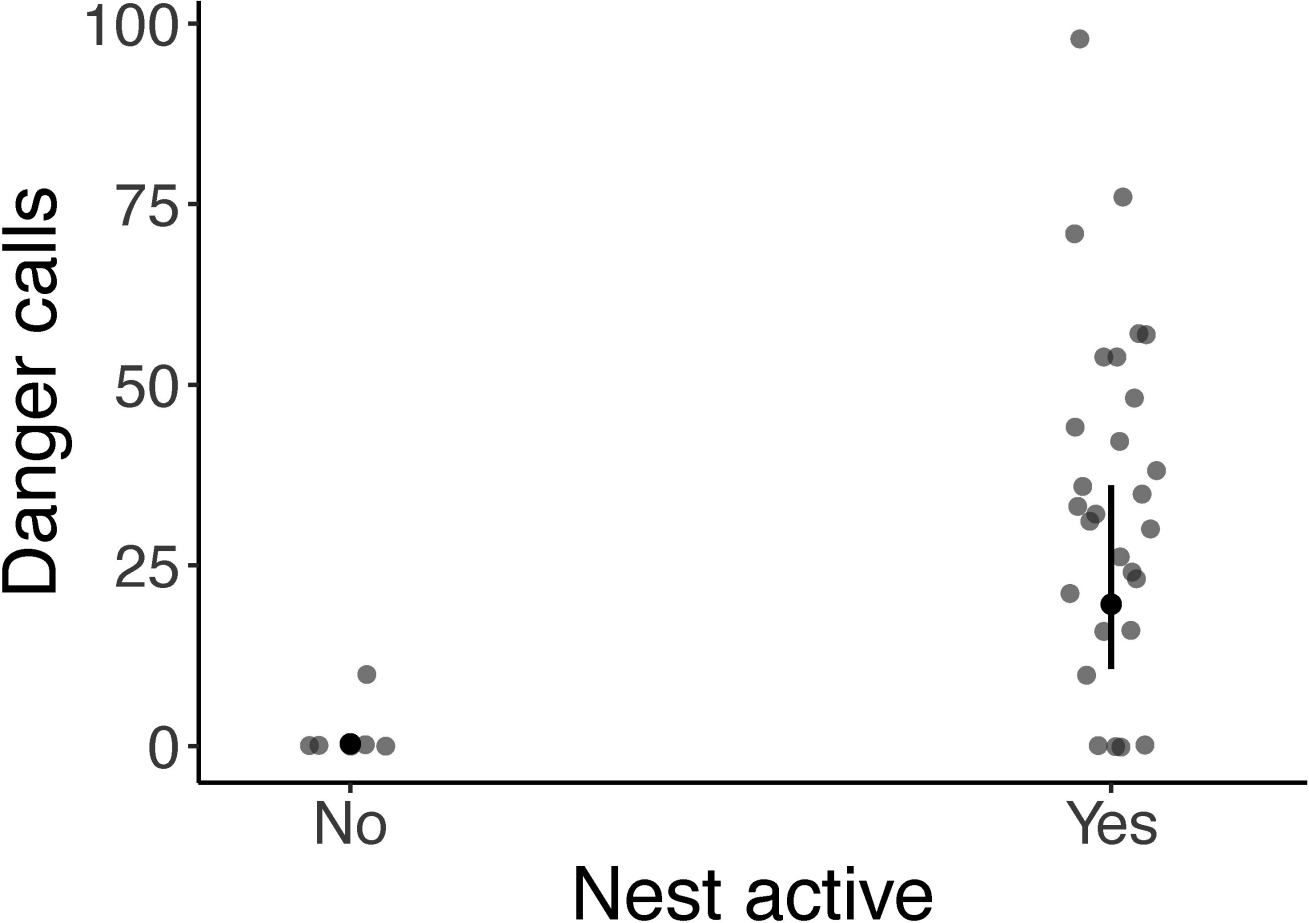
The effect of nest activity (yes = nest active, no = nest inactive) on the number of danger calls produced by superb fairy-wrens per minute in response to a human standing at the nest (*n* = 34). Full model output presented in table 1.

During monitoring, we noted that superb fairy-wrens often gave danger calls when humans or terrestrial predators (grey currawongs, *Strepera versicolor*, that were walking on the ground, which do not elicit aerial alarm calls) were near active nests. Nest activity (i.e. whether the nest was being actively built or contained offspring) was associated with the number of danger calls. Danger call rates in response to a human standing directly at the nest differed significantly between active and inactive nests (estimate = 2.89 ± 0.33, *p* < 0.001; table 1, figure 2). At active nests with a human within 1 m, birds produced an average of 13.6 ± 1.4 danger calls per minute. No calls were recorded at five recently inactive and empty nests. At one such nest, however, the breeding pair gave 10 danger calls in 1 min while constructing a new nest less than 20 m away. However, we note the low sample size of inactive nests in this study.

**Table 1.** Poisson GLMM of the number of danger calls per minute in relation to nest activity (active = yes or inactive = no) and group size in superb fairy-wrens (*n* = 34). The random effect territory ID explains 0.65 ± 0.81 of the variance. Bold values indicate statistical significance *p* < 0.05.

	estimate	s.e.	z-value	*p*
intercept	0.26	0.38	0.689	0.491
active [yes]^a^	2.89	0.33	8.677	**<0.001**
group size	0.17	0.23	0.723	0.470

^a^ Reference category set to no (nest inactive)

### 3.3. Call type in relation to nest stage and proximity

At active nests and in response to human approach to the nest, we recorded 2884 danger calls, 0 aerial alarm calls and 3664 terrestrial alarm calls from 37 recordings at 23 nests across 17 superb fairy-wren territories. The average number of calls per minute, categorized by stage and threat distance, is presented in electronic supplementary material, table S1.

**Figure 3.**
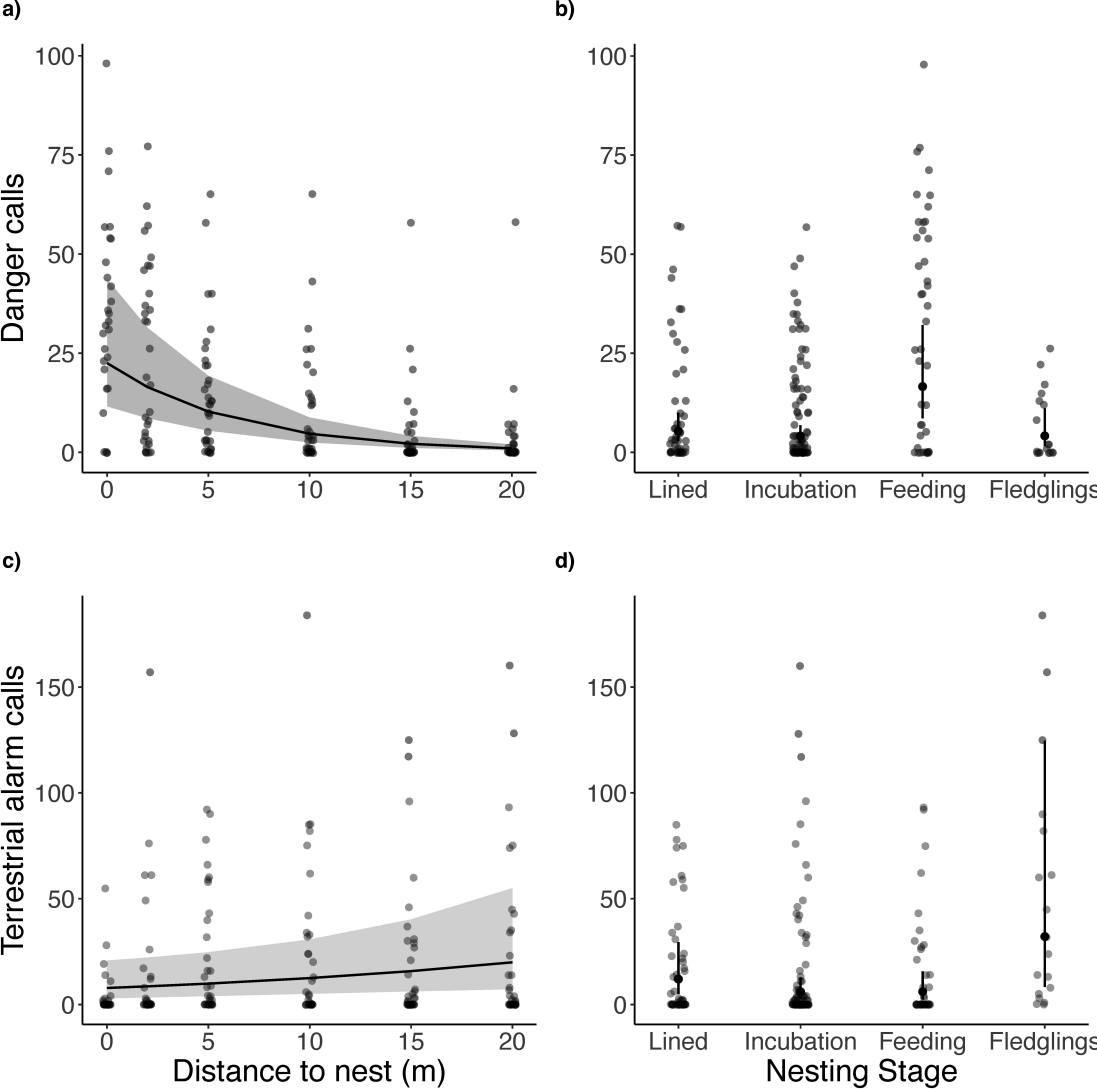
Number of danger calls and terrestrial alarm calls produced per minute in relation to (a, c) threat distance to the nest and (b, d) nesting stage in superb fairy-wrens (*n* = 37 recordings from 23 nests). Full model output presented in table 2. None of the birds produced aerial alarm calls during a standardized human approach to the nest (see §2).

The number of danger calls per minute increased significantly as threat distance to the nest decreased (*χ*² = 97.64, *p* < 0.001; electronic supplementary material, table S2a, figure 3a) and varied across nesting stages (*χ*² = 16.27, *p* < 0.001). More danger calls were produced during the chick feeding stage compared with the nest-lining (post hoc estimate = 1.13 ± 0.42, *p* = 0.038) and incubation stages (estimate = 1.39 ± 0.36, *p* < 0.001; electronic supplementary material, table S3, figure 3). No significant differences were found between other stages (electronic supplementary material, table S3). Group size had no effect on danger call rate (*χ*² = 0.006, *p* = 0.937, electronic supplementary material, table S2).

In contrast to danger calls, the number of terrestrial alarm calls did not differ significantly between nesting stages (*χ*² = 4.79, *p* = 0.188; electronic supplementary material, table S2b). However, there was a marginal trend towards fewer alarm calls with decreasing distance to the nest (*χ*² = 3.33, *p* = 0.068) and increasing group size (*χ*² = 3.29, *p* = 0.070).

**Figure 4.**
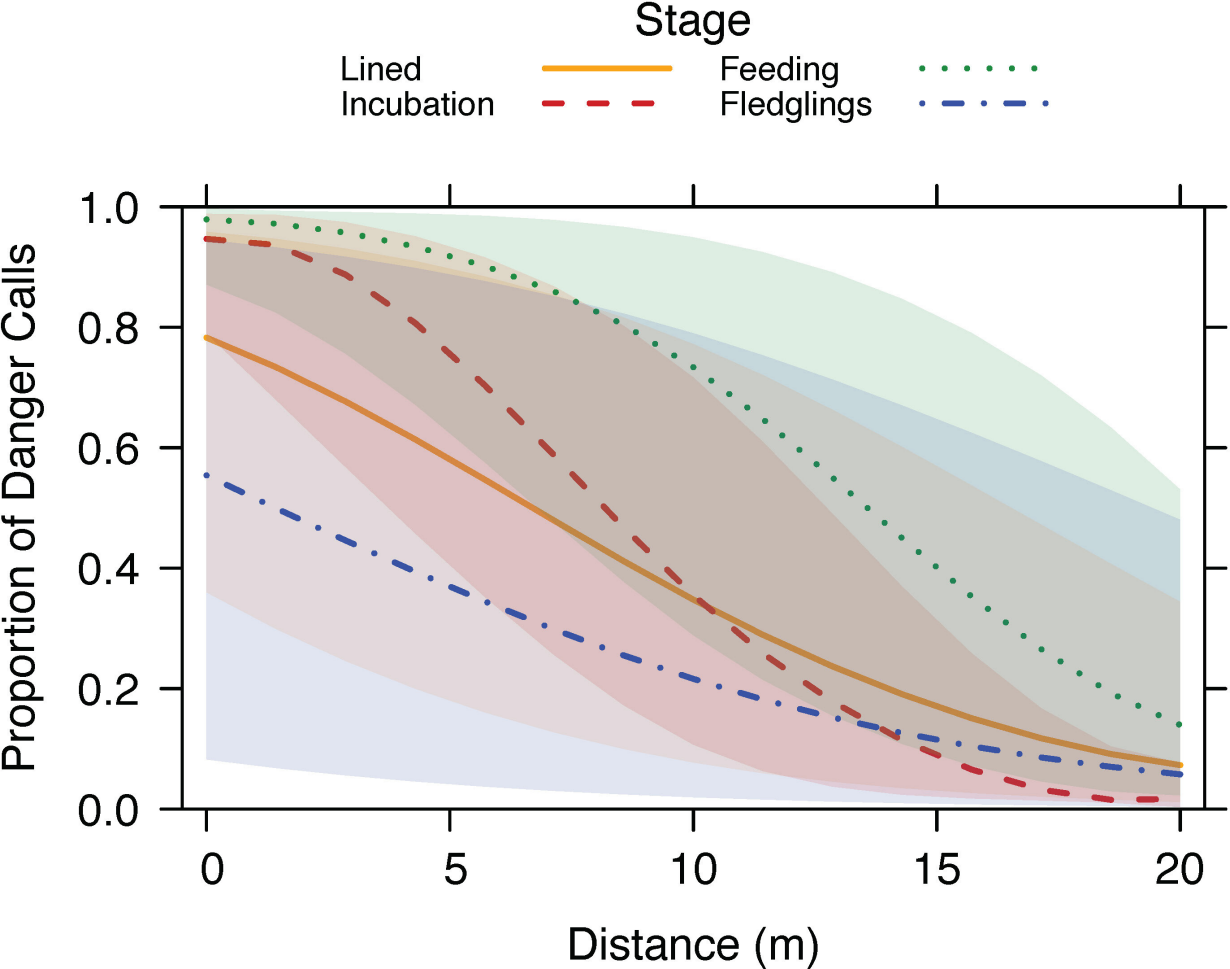
The proportion of danger calls to terrestrial alarm calls produced by superb fairy-wrens in relation to human distance from the nest and nesting stage. Full model output presented in table 2. None of the birds produced aerial alarm calls during a standardized human approach to the nest (see §2).

There was a significant interaction between threat distance to nest and nesting stage on the proportion of danger calls to terrestrial alarm calls (*χ*^2^ = 55.47, *p* < 0.001) (table 2). When the threat was close to the nest, the highest proportion of danger calls was given during the chick feeding stage, and the lowest proportion during the fledgling stage (figure 4). At larger distances (15–20 m from the nest), the majority of calls given were terrestrial alarm calls, regardless of nesting stage. There was no effect of group size on the proportion of danger calls produced (table 2).

**Table 2.** Binomial GLMM of the effect of threat distance, nesting stage, group size and the interaction between threat distance and nesting stage on the proportion of danger calls to alarm calls produced by superb fairy-wrens (*n* = 37 recordings at 23 nests). Random effects territory ID and recording ID explain 3.36 ± 1.83 and 5.04 ± 2.25 of the variance, respectively. Bold values indicate statistical significance *p* < 0.05. No aerial alarm calls were produced during the human approach trials.

	estimate	s.e.	*z*-value	*χ* ^2^	d.f.	*p*
intercept	1.25	0.98	1.28			
threat distance	−2.00	0.11	−18.95	786.69	1	**<0.001**
stage [fledglings]^a^	−2.41	1.51	−1.60	4.31	3	0.230
stage [incubation]^a^	−1.56	0.92	−1.69			
stage [lined]^a^	−1.72	1.13	−1.53			
group size	0.80	0.67	1.19	1.43	1	0.233
threat distance × stage [fledglings]^a^	0.94	0.23	4.15	55.47	3	**<0.001**
threat distance × stage [incubation]^a^	−0.45	0.17	−2.65			
threat distance × stage [lined]^a^	0.65	0.16	4.14			

^a^ Reference category set to feeding

### 3.4. Playback experiment

There was no significant interaction between stimulus type (aerial versus danger call) and playback stage (baseline versus playback) on an individual’s behavioural response to call playback: either time spent within 10 m of the nest or minimum distance to the nest (electronic supplementary material, table S4). Birds spent more time within 10 m of the nest during playback (estimate = 1.17 ± 0.42, *p* = 0.005) and during danger call playback regardless of whether it was the baseline or the playback stage (estimate = 1.08 ± 0.45, *p* = 0.015). Birds responded more strongly to the second playback experiment irrespective of call type (time spent within 10 m: estimate = 1.02 ± 0.26, *p* < 0.001; minimum distance: estimate = 1.49 ± 0.48, *p* = 0.002).

**Figure 5.**
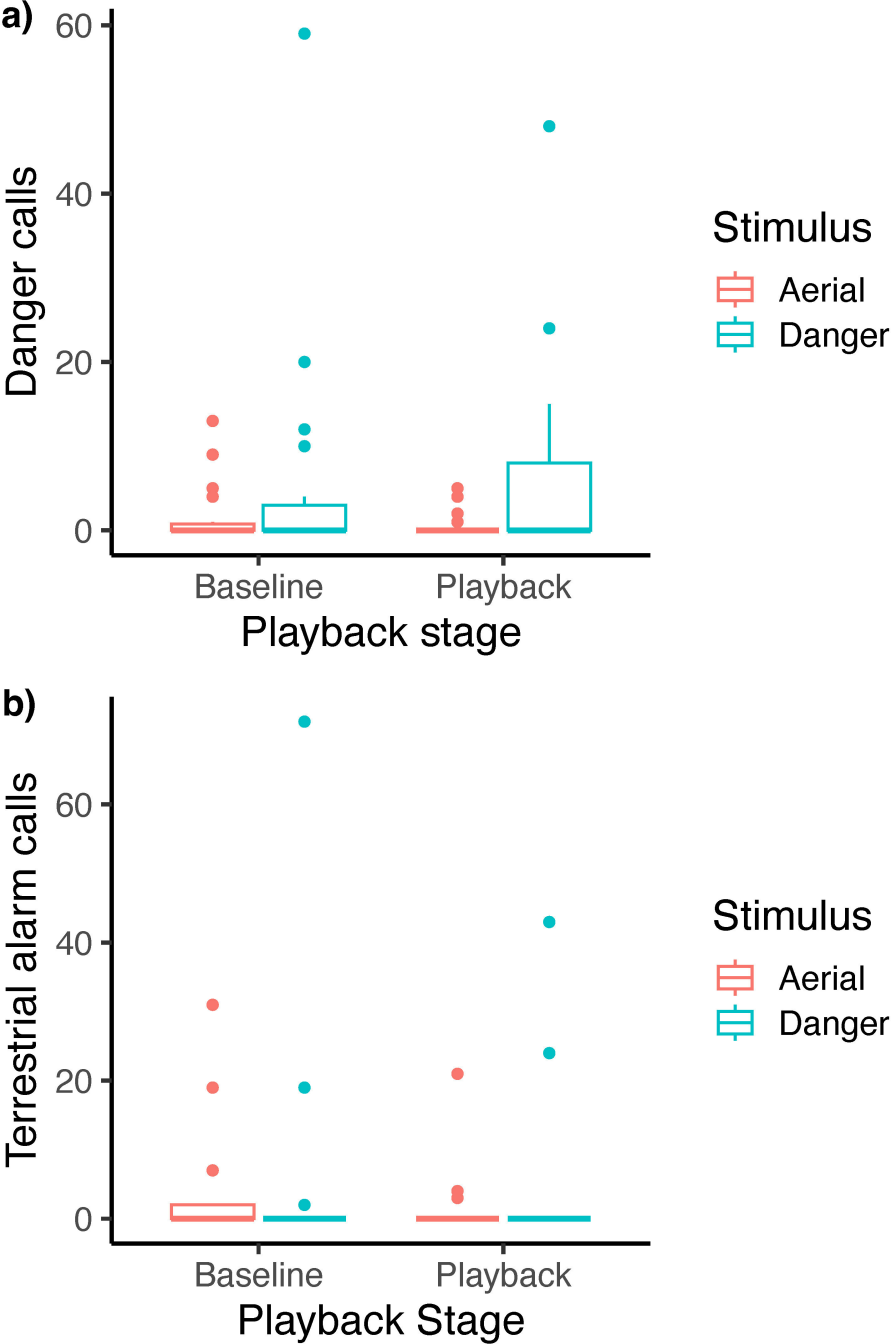
The difference in vocal response of superb fairy-wrens based on playback call type (aerial alarm versus danger call), measured as the number of (a) danger calls and (b) terrestrial alarm calls produced by the group. Full model output is presented in table 3.

Groups produced more danger calls in response to danger call playback than to aerial alarm playback (call type × stage: estimate = 1.13 ± 0.36, *p* = 0.002; table 3a; figure 5a). Groups produced significantly fewer danger calls during the aerial call playback compared with baseline (post hoc estimate = 0.98 ± 0.34, *p* = 0.020). Groups also produced significantly more danger calls in the second playback trial compared with the first (estimate = 0.99 ± 0.22, *p* < 0.001; table 3a). The number of terrestrial alarm calls did not differ between broadcast of danger calls or aerial alarm calls, playback stages or their interaction (table 3b; figure 5b). There were no aerial alarm calls produced during any stage of the playback experiment.

**Table 3.** Results of the experimental broadcast of call type (aerial, danger), stimulus stage (baseline versus playback), presentation order and group size (number of birds within 15 m) on playback response in superb fairy-wrens: (a) number of danger calls produced by the group and (b) number of terrestrial alarm calls produced by the group. Playbacks were done between day 7 and 9 of the incubation phase. Random effects explained (a) territory ID: 1.21 ± 1.1; trial ID: 2.89 ± 1.7; and (b) territory ID: <0.01 ± 0.01; trial ID: 5.00 ± 2.2 of the variance. (a) Poisson GLM, (b) negative binomial GLM. Bold values indicate statistical significance *p* < 0.05.

*a) danger calls*				
	estimate	s.e.	z-value	*p*
intercept	−0.26	1.98	−0.13	0.894
call type^a^	0.88	0.25	3.54	**<0.001**
stage^b^	−0.98	0.34	−2.90	**0.004**
order^c^	0.99	0.22	4.51	**<0.001**
group size	−0.29	0.68	0.42	0.672
call type × stage	1.13	0.36	3.10	**0.002**

*b) terrestrial alarm calls*				
intercept	0.32	2.98	0.11	0.915
call type^a^	−1.34	1.56	−0.86	0.389
stage^b^	−2.02	1.34	−1,51	0.132
order^c^	2.10	1.17	1.80	0.072
group size	−0.20	0.92	−0.22	0.830
call type × stage	0.35	1.95	0.18	0.857

^a^ Aerial used as reference category

^b^ Baseline used as reference category

^c^ Order (1) used as reference category

## 4. Discussion

We describe and investigate an alarm call in the widely studied superb fairy-wren, the ‘seet’ danger call. Danger calls are acoustically distinct from previously described aerial and terrestrial alarm calls, with frequency bandwidths similar to the aerial alarm call (figure 1). The highest number and proportion of danger calls were produced during the chick feeding phase, but this call was given during all nest building and reproductive phases, including before egg laying. The lowest proportion of danger calls was given after the chicks had fledged. Adult superb fairy-wrens also showed distinct vocal responses to playback of danger versus aerial alarm calls, despite the two call types occupying similar frequency ranges. When danger calls were broadcast near the nest, individuals produced more danger calls than in response to aerial alarm calls. Throughout our year-round monitoring and observations in non-nesting contexts, we rarely observed danger calls being produced, although it remains possible that the danger call is used outside of nesting contexts. We did observe groups producing danger calls when a human was near an injured, non-nesting adult wren (LK Common, personal. observation). We propose that the ‘seet’ danger call functions to alert family members and conspecifics to the presence of a threat near vulnerable, immobile individuals—such as eggs or chicks in the nest.

Danger calls were high-pitched, with a narrow bandwidth, and produced as single elements. These characteristics make the caller harder to spatially localize compared with terrestrial alarm calls [2,61,62]. Both terrestrial and aerial alarm calls in the superb fairy-wren have rapid frequency modulation [32,33], a feature that is not found in the danger call. The acoustic properties of the terrestrial alarm call are conducive to localization [62]. Indeed, the terrestrial alarm call generally functions as a mobbing call [23], and group members are often conspicuous and easily located while giving terrestrial alarm calls (LK Common, personal observation). Conversely, group members also generally approached the threat but remained hidden in shrubs while producing danger calls (LK Common, personal observation). The opacity of danger call localization would benefit the caller who can alert group members about a predator’s presence without revealing the location of the nest or vulnerable conspecifics to the predator. ‘Seet’ calls in other fairy-wren species probably function to increase vigilance in group members when in the presence of predators [38,39]. The danger call may therefore function as a precursor to mobbing or rodent running, a distraction display where individuals run along the ground away from the nest/individual in a rodent-like posture, given in response to distress calls [35]. However, although the rodent run is performed when a nestling, fledgling, or adult is in distress, it is rarely performed during incubation and has not been documented during nest building (LK Common and D Colombelli-Négrel, personal observation) and was not observed during data collection for this study. There is no current observation supporting the view that the danger call during early nesting stages is associated with the rodent run. Alternatively, given its function to increase vigilance, the danger call may increase monitoring of predator activity near the nest to facilitate rapid renesting if, for example, the nest is found by a predator in early (pre-egg laying) nesting stages, as predator nest detection at these stages may not leave physical evidence. Therefore, the detection of threats near the nest and the dissemination of that information through the group could assist a female in her decision of whether to abandon and renest or continue the nesting attempt. Fairy-wrens will renest quickly following nest failure when prevailing conditions are suitable, often starting to build a new nest the day after predation (LK Common, personal observation). Future research could investigate whether some females abandon nest building when a current nest has been detected by predators, as signalled by the danger call. Defending the nest against a predator by mobbing or producing mobbing calls can be costly [63,64], and beginning or delaying renesting following predator detection carries other costs. Understanding how the danger call functions during predation events and influences risk-taking, communication and reproductive investment decisions will deepen our knowledge of how animals perceive and make decisions in varying risk environments.

The intended receiver of the danger call is not currently known, although there is some evidence that the call is directed to other adult group members, rather than offspring. Randler [16] described a breeding-season-specific call similar in structure to the danger call in Cyprus wheatears (*Oenanthe cypriaca*) named alarm call type II. Call type I in this species, somewhat similar to a terrestrial alarm call in fairy-wrens, was given at a higher rate during incubation, with nestlings and with young fledglings, whereas the type II alarm call was given almost exclusively during the fledgling phase and not at all during the incubation phase [16]. Therefore, it was proposed that type I calls probably function as within-pair communication and type II function as parent–offspring communication [16]. In stonechats (*Saxicola torquata*), ‘whit’ calls were given when a human intruder was near the nest [65]. ‘Whit’ call rate increased dramatically after hatching and peaked after fledging, with an absence of calling during egg laying and low rates during incubation [65]. This call also significantly reduced nestling begging in a playback experiment, suggesting that it functions as parent–offspring communication. These two studies differ from our results, as we found that danger calls were given even during nest building and incubation, therefore under conditions without offspring (empty nest) or with offspring (eggs) that are unable to modify their behaviour based on this signal [66]. This suggests that the danger call may be directed towards adult receivers to inform decisions about current (e.g. continue nesting or abandon) or future reproductive investment (e.g. renest in the same area or relocate), rather than serving as a form of parent–offspring communication. Whether embryos adjust their heart rate to danger calls, as seen in other bird species [47,67], remains to be investigated. In addition, the lowest proportion of danger calls was produced during the fledgling stage, when offspring were highly vulnerable but also able to perform antipredator behaviour [3,68,69]. With our current dataset, we were unable to test whether fledgling age influenced danger call rate; however, we predict that groups with older, more mobile, fledglings would produce fewer danger calls and more terrestrial alarm calls in response to threats, an area worthy of further investigation. Future research should also investigate whether the danger call elicits a response in offspring, or whether it more strongly influences current and/or future reproductive investment decisions by attending parents and helpers. Examining offspring responses to danger calls will help clarify the potential for different functions depending on the call’s intended receivers.

There is significant evidence across species, and in fairy-wrens specifically, that calls encode information on predator characteristics [69] and that calls can elicit nest defence behaviour [34]. There is little to no information on whether calls can encode information about receiver or prey characteristics [21]. In this study, the rate of danger calls was the lowest as the offspring became mobile, i.e. during the fledgling phase, despite the high vulnerability of young fledglings [68]. In contrast, the proportion of terrestrial alarm calls was highest during the fledgling phase and was used during experimentally induced fledgling defence [69]. Interestingly, in two cases, danger calls were given by three adult group members when a human was near an injured adult female with impaired mobility, with no observation near hundreds of non-injured adults (LK Common, personal observation). The rate of danger calls may therefore refer to the urgency of the threat not only as it relates to predator distance from a nest but also to conspecific or group-member vulnerability. As chicks grow, they change their appearance, which may provide attending parents with cues about profitable nest defence investment tactics [70]. In *Acrocephalus* warblers, for example, the inflection point during chick growth corresponds with the day chicks open their eyes and also corresponds with the day that parents begin to predictably produce alarm calls in the face of a threat to nesting contents [70]. Perhaps fairy-wrens integrate multiple cues about the vulnerability of nest contents and/or group members, which could explain some of the variance in danger call production. However, the context in which the calls are given and the cues that elicit them are not yet understood, especially if the function is to inform the risk context of reproductive decisions. At present, our data do not indicate a clear association between danger call production and vulnerability *per se*, despite anecdotal observations of such calls being given near a wounded adult and experimental evidence of calls being produced at the highest rate near immobile nestlings.

Our study has some limitations that constrain the conclusions we can draw about call function. Because the playback experiment did not include terrestrial alarm calls, we were unable to make a full comparison across the three alarm call types. Fairy-wrens respond to playback of terrestrial alarm calls, also known as mobbing calls, with increased vigilance and by approaching the speaker, and respond to aerial alarm calls by fleeing [32,71]. It remains possible that the danger call functions as a precursor to mobbing or overlaps functionally with the terrestrial call, and future work is required to test whether the two are referentially distinct, as we suspect. Additionally, as we aimed to control the urgency of the calls, our playback consisted of only a single call per second for both the aerial and danger call playback. Aerial calls are commonly given in bouts, with increasing calls per bout indicating higher urgency [36]. In our design, the aerial call playback probably represents the lowest urgency, and the rate more closely follows the natural calling rate of danger calls, which could explain why we did not see a significant individual-level behavioural difference between call playbacks. The aerial alarm calls used in the experiment were from unfamiliar individuals, whereas danger calls were recorded from familiar callers (at the territory level). To our knowledge, it is not currently known whether there are signals of individuality encoded in aerial and danger calls and whether individuals discriminate based on these signals. Fairy-wrens have been found to respond more strongly to alarm songs [37] and terrestrial alarm calls [32] of familiar birds compared with unfamiliar birds, so caller identity may also have affected the response to aerial and danger calls seen in this study. Still, fairy-wrens were quieter following aerial alarm call playback, giving more danger calls in response to the danger call playback. Future research comparing behavioural responses with all three alarm call types, at different urgencies, across breeding and non-breeding contexts and with known caller identity, will be essential to clarify call functions.

Despite these limitations, several lines of evidence support the view that the danger call is a distinct vocalization type. Acoustically, it is a single, high-pitched, narrow-band downward sweep, unlike the rapidly modulated bouts of terrestrial calls or the more inflected aerial calls; acoustic analysis confirmed that the three call types can be discriminated. Danger calls were typically given singly and at slower rates, contrasting with the fast, repeated structure of terrestrial mobbing calls. Although aerial and danger calls share a similar frequency range, aerial calls were never produced when humans approached nests, whereas danger calls were frequent in that context, suggesting functional separation despite spectral overlap. Finally, the structural features of the danger call—high frequency and low modulation—make it harder to localize, which could conceal the nest while still warning conspecifics, in contrast to conspicuous terrestrial mobbing calls that recruit group members. Taken together, these acoustic, contextual and functional features provide evidence that the danger call is a novel addition to the superb fairy-wren repertoire, with a vigilance or warning function distinct from both aerial and terrestrial alarm calls.

## 5. Conclusion

‘Seet’ calls have been described in other fairy-wren species [38,39]. Here, we term this ‘seet’ call as a ‘danger’ call in superb fairy-wrens and investigate its function. The danger call is regularly produced during the breeding season in response to terrestrial threats near active nests that are being built or contain eggs or chicks and, occasionally, but much less frequently, in the presence of young fledglings. Therefore, this call is both situation-specific (i.e. functionally referential) and, since it potentially encodes information on threats to current reproductive success, urgency-based [72]. To demonstrate functional reference, both the signaller’s and receiver’s perspectives must be considered; the call should elicit a distinct response in the receiver compared with other alarm calls [4,73]. We found that, although similar in their frequency range, the danger call elicited a distinct vocal response compared with the aerial alarm call during the incubation phase of nesting, although this finding is preliminary. This call offers a novel perspective for understanding threat assessment and communication about risk perception during the nesting phase.

## Data Availability

Data and R code used for analysis are available as supplementary material [74].
